# Personalized Optical Designs and Manipulating Optics: Applications on the Anterior Segment of the Eye

**DOI:** 10.1155/2020/9586062

**Published:** 2020-02-25

**Authors:** Pablo Pérez-Merino, Damian Siedlecki, Laura Remón, Maria Vinas, Jorge L. Alió, Jos J. Rozema

**Affiliations:** ^1^Department of Ophthalmology, Instituto de Investigación Sanitaria Fundación Jiménez Díaz, Madrid, Spain; ^2^Wroclaw University of Science and Technology, Wroclaw, Poland; ^3^Departamento de Física Aplicada, Universidad de Zaragoza, Zaragoza, Spain; ^4^Instituto de Óptica, Consejo Superior de Investigaciones Científicas (CSIC), Madrid, Spain; ^5^Vissum Corporacion Oftalmologica, Alicante, Spain; ^6^Department of Ophthalmology, Antwerp University Hospital, Edegem, Belgium; ^7^University of Antwerp, Antwerp, Belgium

The image-forming properties of the eye can be described in terms of wave aberration. Understanding the link between aberrations and the anterior segment geometry is therefore of crucial importance for (i) comprehending how the eye works, (ii) modelling the optics of individual eyes, (iii) optimizing optical solutions, or (iv) designing surgical strategies. The eye has many innate adaptations that minimize optical aberrations. In most normal young eyes, the magnitude of aberrations of the cornea is significantly larger than for the whole eye, indicating a significant role of the crystalline lens in compensating corneal aberrations. However, due to geometrical and structural changes, this ocular compensation gets disturbed in different anterior segment conditions, such as keratoconus, presbyopia, or cataract. Keratoconus progressively degrades the corneal shape and, consequently, vision in the adolescence, with a prevalence of 0.05% in the general population. Meanwhile, presbyopia and cataract are conditions related to aging that affect the structure of the crystalline lens, one referring to a loss in accommodative amplitude (presbyopia) and the other to a progressive loss of transparency (cataract). Presbyopia affects 100% of the population older than 45 years of age, impairing reading and near work activities as a result of the loss of the eye's crystalline lens ability to focus at near objects, whereas cataracts affect more than 50% of the population over 75. Furthermore, the prevalence of myopia has alarmingly increased in recent years, especially in the developing economies of the East Asian area, making the identification of the optical changes in the eye associated with myopia particularly important.

Given the large numbers of potential patients for optical correction of presbyopia and cataract, managing keratoconus, or slowing down the progression of myopia, the potential impact of new solutions based on personalized optical designs is undoubtedly tremendous and will offer both remarkable opportunities and challenges in a wide range of anterior segment applications. Combining the technological advances in aberrometry and three-dimensional anterior segment imaging techniques with dedicated ray-tracing and processing tools will allow building patient-specific eye models for the selection of the contact and/or intraocular lens design that provides the best optical quality [1–3].

Furthermore, a better understanding of the optical corrections and how they interact with the patient's optics will help clinicians with selecting the correction that optimizes the visual performance in their patients, for example, using visual simulators that allow the patient to experience the difference in visual performance between monofocal or different multifocal solutions [4]. With the recent expansion of presbyopia-correcting contact/intraocular lenses by the many variations in design (diffractive and refractive, or new class of extended depth-of-focus lenses), multiple zones, and add power, the ability to satisfy the patient's visual demands has never been greater, possibly leading to unprecedented customized eye treatments. In this special issue, eleven scientific articles and one review article discuss the implications and possible future directions of personalized optical designs and manipulating optics through different applications on the anterior segment of the eye.

It is well established that favourable interactions between low- and high-order aberrations can improve the visual performance [5]. In particular, adding a certain amount of spherical aberration to defocus can improve visual quality over defocus alone. Besides defocus, specific combinations of astigmatism and coma also increase optical quality. Zhang et al. investigated the correlations between spherical aberration, astigmatism, and axial length in a large cohort (6747 eyes) and found that in eyes with low astigmatism axial length was correlated with spherical aberration (e.g., a gradual decrease as axial length increases). In the presence of larger astigmatism (>2D), however, the authors found different tendencies between axial length and spherical aberration that would clearly affect the selection of a toric IOL. Velasco-Barona et al. evaluated the effect of angle kappa and aberrations on the visual performance of two different presbyopia-correcting IOLs (diffractive trifocal: Acrysof IQ PanOptix and AT LISA tri 839MP). The authors did not notice association between angle kappa and the postoperative visual quality. In a different study, Liu et al. provided clinical advice in choosing presbyopia-correcting IOLs by comparing the visual performance between the Echelette Extended Range of Vision (Tecnis Symfony ZXR00) and the diffractive bifocal (Tecnis ZMB00). Regarding IOL power calculation, Fernández et al. described that the postoperative residual refractive error with current IOL power calculation formulas could be associated with an under- or overestimation of the real estimated lens position (ELP). But even if ELP was perfectly predicted, there may still be some postoperative refractive error depending on the axial length. Hence, the authors proposed a fictitious corneal refractive index estimation as an additional method to optimize the IOL power calculation.

The replacement of the crystalline lens by an intraocular lens modifies the chromatic dispersion properties of the eye, depending on the dispersion properties of the IOL material. Moreover, different IOL materials have different tendencies to form small fluid-filled vacuoles (glistenings) within the bulk of the IOL with different effects on light scattering. Spiezio et al. proposed a new methodology based on high-magnification digital microscopy to quantitatively evaluate and characterize such IOL vacuoles using their critical optical characteristics, such as vacuole size, density, shape, and orientation within the IOL material. Elwan et al. showed a new technique for the treatment of primary posterior capsule opacification (PCO) or prevention of postoperative PCO based on a pneumatic technique to perform a posterior capsulorhexis.

Progressive distortion of the cornea in keratoconus leads to abnormal corneal topography, resulting in irregular astigmatism, progressive myopia, and increased high-order aberrations. Although the anterior corneal surface supposes the dominant factor to corneal aberrations, the posterior corneal surface also has a remarkable implication in ocular aberrations. Therefore, an accurate three-dimensional quantification of both anterior and posterior corneal surfaces is critical for managing keratoconus. Velázquez-Blázquez et al. proposed a three-dimensional virtual model of the cornea by means of computational geometry as a novel tool for keratoconic classification. Corneal surface analysis in corneal topographers is usually based on fitting the elevation data to parametric models to obtain the relevant information. Regarding keratoconus classification based on Scheimpflug-imaging surface fitting, Garcerant et al. defined a toric ellipsoid with fixed eccentricity at the thinnest point as the best-performing parameter to discriminate between normal and keratoconus within a myopic population.

In the contact lens field, Wang and Yang described the effect of decentration of the orthokeratology lens on myopia progression. Meanwhile, the review article by Remón et al. proposed novel experimental paradigms for presbyopia correction and myopia control, such as material platforms, optical designs, and computational simulations of bifocal and multifocal contact lenses. In a different study, Carracedo et al. compared the agreement and repeatability of binocular open-field and monocular closed-field wavefront autorefractor systems. The binocular open-field provided better results in terms of spherical equivalent and *J*_0_ and could be an excellent tool to evaluate refractive errors in the clinical practice.

Interestingly, Montagud-Martínez et al. showed a novel concept of using diffractive corneal inlays for presbyopia correction. This design not only surpasses the multifocal performance of the commercial small aperture corneal inlays but also compensates their limitations in terms of degraded contrast sensitivity and stereoscopic acuity.
